# A Review on Triple Antibiotic Paste as a Suitable Material Used in Regenerative Endodontics 

**DOI:** 10.22037/iej.v13i1.17941

**Published:** 2018

**Authors:** Zahed Mohammadi, Hamid Jafarzadeh, Sousan Shalavi, Shapour Yaripour, Farid Sharifi, Jun-Ichiro Kinoshita

**Affiliations:** a *Iranian Center for Endodontic Research, Research Institute of Dental Sciences, Dental School, Shahid Beheshti University of Medical Sciences, Tehran, Iran and Iranian National Elite Foundation, Tehran, Iran; *; b *Dental Research Center, Dental School, Mashhad University of Medical Sciences, Mashhad, Iran; *; c *General Dental Practitioner, Hamedan, Iran; *; d * Department of Oral and Maxillofacial Surgery, Hamedan University of Medical Sciences, Hamedan, Iran; *; e *Dental School, Mashhad University of Medical Sciences, Mashhad, Iran; *; f * Department of Conservative Dentistry, Showa University Dental Hospital, Tokyo, Japan*

**Keywords:** Ndodontics, Intra-canal Medicament, Regeneration, Triple Antibiotic Paste

## Abstract

As the root canal system shows different and complicated anatomies, mechanical instrumentation alone has not the ability to provide a bacteria-free environment in root canals. On the other aspect, necrotic tissue remaining can decrease the effects of root canal irrigants and medicaments and also interfere with the adaptation of root canal fillings to dentin. As a result, certain disinfection and irrigation procedures are required to remove the remaining tissues from the root canal area thoroughly and also be able to eliminate the microorganisms. Triple antibiotic paste (TAP) containing metronidazole, ciprofloxacin and minocycline has been proposed as a root canal medicament due to its antimicrobial effects in endodontic regenerative procedures. The purposes of this review were to determine the properties of TAP drugs and to evaluate the efficiency of TAP on the root canal disinfection, in primary and permanent teeth, along with its affection in regeneration/revascularization procedures. The biocompatibility and disadvantages of this medicament were also discussed.

## Introduction

Animal and clinical studies have proven the prominent effects of microorganisms in development and perpetuation of pulpal/periapical diseases [1-3]. The elimination of infected root canal microorganisms takes a complicated process. Various evaluations have been demonstrated to decline or eliminate the microbial populations such as different various instrumentation techniques [4], variable irrigation regimens [5, 6], and intra-canal medicaments [7, 8]. It has been proven that mere mechanical instrumentation is not sufficient in making a bacteria-free area in canal system by self which is predictable considering the complex anatomies of the root canal space [9]. On the contrary, there are various *in vivo *and *in vitro* studies which confirm the inefficiency of mere mechanical instrumentation to prepare all portions of root canal walls thoroughly [10] and complete elimination of microorganisms [11]. Certain disinfection procedures are required to be able to eliminate the microorganisms from the canal [12-15]. A suitable material for using as intra-canal medicament seems to be antibiotic. Triple antibiotic paste (TAP) containing metronidazole, ciprofloxacin and minocycline has been proposed as a root canal medicament due to its antimicrobial effects in endodontic regenerative procedures. The purposes of this review were to determine the properties of TAP contents and to evaluate the efficiency of TAP on the root canal disinfection, in primary and permanent teeth, along with its affection in regeneration/revascularization procedures.

***Rationales in local application of antibiotics***

Although systemic antibiotic therapy has proven useful in dental surgical and non-surgical procedures, it also comes with some complications such as various side effects (allergic reactions or toxicities) and the development of resistant strains of microbes. Besides, going through a systemic antibiotic therapy depends on so many factors, including the patient’s compliance in taking a specific dosage regimen, the absorption of these drugs by the gastrointestinal system, the transportation *via* the blood circulatory system in order to get to the infected area which implies the medication-required area having a proper blood supply which is no longer available in teeth with necrotic pulps, a pulpless and infected canal or a root-filled tooth that has become infected. As a result, local application of antibiotics within the canal may be a more effective mode for delivering the drug [12-14].

***Rationales in combining the antibiotics***

Considering the polymicrobial nature of tooth infection, single empirical antibiotics are not able to provide a bacteria-free zone in the canal. In addition, using non-specific antibiotic therapy could result in the destruction of normal bacterial flora which allows residual virulent microorganisms to repopulate the canal. As a result, it is essential to use a combination of antibiotics against all endodontic pathogens to prevent microbial resistance [15].

***Tetracycline***

Tetracycline, including tetracycline-HCl, minocycline, demeclocycline and doxycycline are a group of broad-spectrum antibiotics effective against a wide range of microorganisms. Tetracycline is in the bacteriostatic subgroup of antibiotics. This could be one of the advantages of this subgroup for its safety, because when the bacterial cells are not lysed, there will not be any antigenic byproducts released in the infected area (such as endotoxins) [15]. In addition, tetracycline possess various unique properties except their antimicrobial action, including the inhibition of mammalian collagenases, which prevents tissue degeneration, and the inhibition of clastic cells, which results in anti-resorptive activities. Naturally, inflammatory diseases such as periodontitis include numerous tissue collagenases, which could be prevented by the mentioned tetracycline’s property, thus leading to enhanced formation of collagen and bone [14-17].

In endodontics, tetracycline has been used to erase the smear layer from instrumented root canal walls, irrigate the retrograde cavities during periapical surgical procedures and as an intra-canal medicament. It is typically used in conjunction with corticosteroids and these combinations have antibacterial, anti-inflammatory, and also anti-resorptive properties, which all can help to reduce the inflammatory reaction in periapical area including resorption mediated by some clastic cells [14].

***Metronidazole***

Metronidazole is a nitroimidazole compound which exhibits a broad spectrum of activities against protozoa and anaerobic bacteria. Since it is famous for its affective antimicrobial activities against anaerobic cocci as well as gram-negative and gram-positive bacilli, it has been used widely in the periodontology in both systemic and local forms. Metronidazole destroys bacteria cells by permeating their membrane and then binding to the DNA, disrupting the helix structure and causing a very rapid death. It has been shown that metronidazole is effective against anaerobic bacteria but not in aerobic bacteria and it prevented the growth of all obligate anaerobes tested and is more effective against two of the strains in comparison with the calcium hydroxide [18-20].

In addition, one study performed by Lima *et al.* [21] on the effectiveness of antibiotic-based or chlorhexidine-based medications in eliminating *Enterococcus faecalis *biofilms, represented significant differences between the tested formations. The combination of ciprofloxacin and metronidazole had a remarkable effect in decreasing the number of 1-day old bacterial biofilm [21]. However, metronidazole had no effect on improving the disinfection of biofilms when added to Kerr Pulp Canal Sealer EWT [22]. Another study showed 97% healing when metronidazole-chlorhexidine solution was applied for the treatment of chronic apical periodontitis [23].

Gao *et al. *[24]evaluated a sustained release delivery gutta-percha point containing metronidazole for root canal disinfection and determined the drug concentration *in vitro* and the time that the device maintained an effective drug concentration. The results represent that a remarkable concentration of metronidazole was released over more than 10 days. On the 10th day, 33.13 microgram/mL of metronidazole was released which exceeded the minimum inhibitory concentration of metronidazole.

In another evaluation of the disinfection of dentinal tubules using 2% metronidazole gel, 2% chlorhexidine gel, bioactive glass, and calcium hydroxide showed that chlorhexidine gel had the best disinfection effect and after that, metronidazole gel were more efficient than bioactive glass and calcium hydroxide [22].

***Ciprofloxacin***

Ciprofloxacin is a second-generation fluoroquinolone antibiotic [25]. Its range of effect includes most strains of bacterial pathogens responsible for gastrointestinal, respiratory, urinary tract, and abdominal infections, including *Escherichia coli*, *Legionella pneumophila,* *Haemophilus influenzae, Klebsiellap neumoniae*, *Proteus mirabilis, Moraxella catarrhalis, Pseudomonas aeruginosa*, methicillin-sensitive but not methicillin-resistant *Staphylococcus aureus*, *Staphylococcus epidermidis, Streptococcus pneumoniae, Enterococcus faecalis*, and *Streptococcus pyogenes*. Ciprofloxacin and other fluoroquinolones are being used for this wide broad spectrum of activities, their availability in both oral and intravenous formulations and their excellent tissue penetration [26].

***Combination of antibiotics***

According to the various species of bacteria in an infected root canal, single empirical antibiotic does not seem to be enough in disinfecting a root canal [14]. A non-specific antibiotic therapy will only result in suppressing the natural microbial flora, and an opportunity for the persistent, virulent residual bacteria to repopulate the canal space. Therefore, in order to eliminate the canal pathogens thoroughly and break the resistance of the virulent bacteria, using a combination of antibiotics is necessary. The first experience of using antibiotics in endodontics was reported by Grossman which was a paste so called “PBSC” or “polyantibiotic past”. PBSC was a mixture of penicillin, streptomycin, bacitracin and caprylate sodium. Penicillin was effective on gram-positive organisms, streptomycin for gram-negative organisms, bacitracin for penicillin-resistant strains and caprylate sodium to target yeasts [27]. 

***Applications of TAP***

**Root canal disinfection**

As mentioned before, infections of the root canal system are considered to be polymicrobial consisting of both aerobic and anaerobic bacteria [28]. Therefore, a single antibiotic therapy may not be sufficient to handle canal’s infection for its complexity. A combination of antibiotic is essential to address the diverse flora encountered. It might also decrease the development chance of the resistant bacteria strains. The most practical combination in order to reach a sufficient result is TAP [29]. TAP containing metronidazole, ciprofloxacin and minocycline has been proposed as a root canal medicament due to its antimicrobial effects in endodontic regenerative procedures.

Sato *et al.* [30] evaluated the potential of this paste to eliminate bacteria in deep layers of root canal dentine *in situ*. According to the results, no bacteria were extracted from the infected dentin within the 24 h after the application of the drug combination apart from one case in which a few bacteria were recovered. In addition, the use of Rifampin proved to increase the efficiency of this mixture [31]. It is also proved that TAP has better results in decreasing colony forming units in comparison with calcium hydroxide [32]and it can be used safely and without any long term effect on microleakage of sealing materials such as MTA [33]. 

A case report was presented about an immature mandibular second premolar with a pulpless, infected canal with periapical involvement and a sinus tract. In this case, instead of performing the routine treatment protocol and apexification, two types of antibiotics (metronidazole and ciprofloxacin) were applied to the canal which was left empty after the application. The radiographic findings showed the initiation of apical closure 5 months after the antimicrobial procedure was done. Thickening of the root dentine and complete apical closure was confirmed 30 months after the treatment, which indicated the revascularization potential of a young permanent tooth pulp in a bacteria-free root canal condition [34]. 

An evaluation of the efficacy of TAP in disinfection of immature dog teeth with apical periodontitis showed a significant reduction in mean colony count with the application of TAP [29] as well as another study done on rat molars which resulted in efficient disinfection and root formation [35]Another study which was performed to test the efficiency of TAP-mimic scaffolds on elimination of *Actinomyces naeslundii* biofilm, the main bacteria of traumatized permanent teeth with necrotic pulps, showed that they hold significant ability in eradication of bacterial biofilm, a critical step in regenerative endodontics [36]. In another study, in a comparison between TAP, photo-activated disinfection and calcium hydroxide on disinfecting the root canal, 15% failure for calcium hydroxide and 5% failure for TAP and no failure for photo-activated disinfection were observed, which concludes that photo-activated disinfection is more efficient in disinfecting the root canal [37].

Low concentrations of TAP such as 1, 0.1 and .01 mg/mL possess the ability to eradicate *Enterococcus faecalis* colonies with fewer side effects such as no negative effect on the viability of stem cells of the apical papilla compared to high concentrations such as 10, 100, and 1000 mg/mL [38].

***Regeneration/Revascularization***

TAP is effective in disinfecting necrotic infected pulps, and it creates a suitable environment for vital tissue regenerative processes. In a restrospective study, Bose *et al.* [39] got to the result that regenerative endodontic treatment with TAP and calcium hydroxide has more significant effects in increasing the root length than either the non-surgical root canal treatments or MTA apexification. The dentin thickness increase percentage was the highest when TAP was applied during the treatment compared with the calcium hydroxide or formocresol. The position of calcium hydroxide also influenced the outcome. The calcium hydroxide had better results when applied to the coronal section of the root canal than beyond it [39]. 

Lovelace *et al.* [40] showed that the evoked-bleeding step in regenerative procedures after disinfection with TAP induces the accumulation of undifferentiated stem cells into the canal space from periapical region. These cells could take part in the pulp regeneration process after effective disinfection. Thus, root canals disinfected with sodium hypochlorite and TAP had a significantly less chance of having a periapical lesion, and higher chances of gaining root length and wall thickness.

***Treatment of Primary Teeth***

Nakornchai *et al.* [41] demonstrated that TAP and Vitapex had significant effects in root canal treatment of infected primary teeth (96% success rate for both materials). At 6 and 12 months the success rate of TAP and Vitapex were 100% and 96%, respectively. Another study, replaced ornidazole with metronidazole in TAP, and got to the result that it had better results in the periods of 3, 6, and 12 months after treatment of infected teeth, for ornidazole had better efficacy, longer duration of action and slower metabolism compared to metronidazole [42].

A study appraised the clinical and radiographic success rates of TAP in non-instrumentation endodontic treatment of primary mandibular molars at 24-27 months postoperatively. The results showed that this protocol had a high success rate but its success rate based on radiographic evaluation at 2-year follow-up was low [43].

Takushige *et al. *[44] evaluated the efficacy of TAP on the clinical outcome of so-called ‘Lesion Sterilization and Tissue Repair’ therapy in primary teeth with periradicular lesions. Clinical symptoms such as, sinus tracts, gingival swelling, induced dull pain, *etc*, disappeared after treatment in all cases except four which had resolution of the clinical signs and symptoms after further treatment when repeating the procedure. The teeth treated successfully, had a normal radiographic view and normal eruption. All cases were evaluated successful eventually with a mean functional time of 680 days, apart from one for congenital reasons.

According to another study, TAP had the ability to treat a horizontal root fracture in a maxillary central incisor [45].

***Biocompatibility of TAP***

A study performed to evaluate the response of rat subcutaneous tissues to TAP, got to the result that it responded as a moderate inflammation to the paste in the first 15 days, which was reduced to mild in 30 days [45].

Ruparel *et al.* [46] evaluated the human stem cells of the apical papilla (SCAP) survival after exposure to different dilutions of TAP, modified TAP, or a double antibiotic paste (metronidazole and ciprofloxacin). Results showed that, in a clinical situation, a 1000 mg/mL solution is necessary to create pasty slurry for antibiotic pastes. The current clinical situations could be risky for the survival of SCAP cells during TAP dressing and consequently to the ultimate result of dentin-pulp regeneration.

Pereira *et al.* [47] in 2014 concluded that comparing calcium hydroxide; TAP induced an exuberant angiogenic and inflammatory response, higher vascular area, and more inflammatory cells. 

***Drawbacks of TAP***

One of the major concerns regarding the use of TAP is tooth discoloration after treatment studies indicated that TAP was associated with the highest amount of discoloration in comparison with the control groups and other antibiotics which was related to minocycline. Resultantly, application of double antibiotic pate (DAP) has been proposed in some instances [48-51]. In other studies, it was sown that applying DAP or TAP for 1 month significantly reduced dentin microhardness [52, 53].

## Conclusion

The elimination of bacteria from the root canal system plays a major and critical role in success of the endodontic treatment. It seems that TAP can be efficiently used for obtaining this purpose. It may be a promising medicament in new endodontics; however, more researches are needed to open new windows to endodontics.
